# Artificial intelligence-aided ultrasound imaging in hepatopancreatobiliary surgery: where are we now?

**DOI:** 10.1007/s00464-024-11130-0

**Published:** 2024-08-19

**Authors:** Mustafa Bektaş, Catherine M. Chia, George L. Burchell, Freek Daams, H. Jaap Bonjer, Donald L. van der Peet

**Affiliations:** 1 Amsterdam UMC Location Vrije Universiteit Amsterdam, Surgery, De Boelelaan 1117, Amsterdam, The Netherlands; 2 Department of Computer Science, Vrije Universiteit Amsterdam, De Boelelaan 1105, Amsterdam, The Netherlands; 3 Amsterdam UMC Location Vrije Universiteit Amsterdam, Medical Library, De Boelelaan 1117, Amsterdam, The Netherlands

**Keywords:** Artificial intelligence, Ultrasound, Hepatopancreatobiliary surgery

## Abstract

**Background:**

Artificial intelligence (AI) models have been applied in various medical imaging modalities and surgical disciplines, however the current status and progress of ultrasound-based AI models within hepatopancreatobiliary surgery have not been evaluated in literature. Therefore, this review aimed to provide an overview of ultrasound-based AI models used for hepatopancreatobiliary surgery, evaluating current advancements, validation, and predictive accuracies.

**Method:**

Databases PubMed, EMBASE, Cochrane, and Web of Science were searched for studies using AI models on ultrasound for patients undergoing hepatopancreatobiliary surgery. To be eligible for inclusion, studies needed to apply AI methods on ultrasound imaging for patients undergoing hepatopancreatobiliary surgery. The Probast risk of bias tool was used to evaluate the methodological quality of AI methods.

**Results:**

AI models have been primarily used within hepatopancreatobiliary surgery, to predict tumor recurrence, differentiate between tumoral tissues, and identify lesions during ultrasound imaging. Most studies have combined radiomics with convolutional neural networks, with AUCs up to 0.98.

**Conclusion:**

Ultrasound-based AI models have demonstrated promising accuracies in predicting early tumoral recurrence and even differentiating between tumoral tissue types during and after hepatopancreatobiliary surgery. However, prospective studies are required to evaluate if these results will remain consistent and externally valid.

**Graphical abstract:**

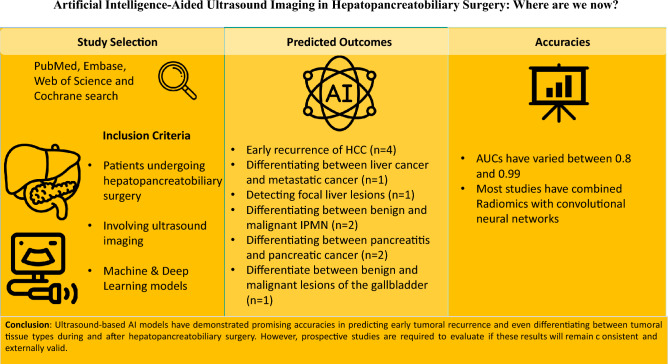

**Supplementary Information:**

The online version contains supplementary material available at 10.1007/s00464-024-11130-0.

Ultrasound imaging has been key in detecting gastrointestinal pathologies for many years. Especially many cholecystitis, appendicitis, pancreatitis, and gallstones cases have been successfully diagnosed with the use of ultrasound [1]. Compared to other imaging modalities, ultrasound has no radiation, does not require patient preparation, and can be performed quickly. In addition, ultrasound can be used to provide diagnostic information, but also serve as a screening instrument [2]. The introduction of endoscopic ultrasound has enabled even more treatment opportunities within gastrointestinal surgery, such as applying angiotherapy and collecting abdominal fluids [3].

However, challenges of ultrasound imaging are still present, such as the inability to detect small tumors or fistulas within the gastrointestinal tract. Visualization of anatomical structures such as the bile or pancreatic ducts is also difficult with ultrasound only, as such structures are overshadowed by luminal gas bubbles or liquids in the gastrointestinal tract [4]. Furthermore, the accuracy of ultrasound imaging is highly dependent on the clinician’s experience and pathology progression [5]. There are many cases in which misdiagnosis has occurred [6], consequently diagnostic accuracies of ultrasound imaging have been reported to fluctuate between 50 and 90% [7].

The current challenges could be overcome by applying AI models on ultrasound imaging. In a typical medical image-based AI model, ultrasound images are exported and converted into specific formats such as jpg or DICOM (Digital Imaging and Communications in Medicine) format. Using software tools, a region of interest (ROI) can be drawn to segmentate the relevant anatomical structure manually or automatically, depending on the tool [8]. Within this ROI, tumoral features are extracted by using radiomics. To build the AI model, machine learning or deep learning methods are selected to combine the extracted features and clinical variables of patients to make predictions. By training on large datasets and recognizing patterns within data, AI models can increase the accuracy of the prediction model efficiently [9]. Using this ability on ultrasound imaging could optimize the diagnostic accuracy of ultrasonography. Along with the benefits of being easily applicable, and quickly accessible, combining AI models and ultrasound might be very promising for detecting and predicting gastrointestinal diseases that require surgery.

Although AI models have been frequently applied within hepatopancreatobiliary surgery, the current status and progress of ultrasound-based AI models have not been evaluated in recent literature. Therefore, this review aims to evaluate the development, validation, and predictive performances of ultrasound-based AI models that have been used in hepatopancreatobiliary surgery.

## Materials and methods

Literature was retrieved and systematically reviewed in conformity with the Cochrane Handbook for Systematic Reviews of Interventions version 6.0 and PRISMA guidelines. This review was registered in the PROSPERO database (CRD42024525032).

### Literature search

A systematic search was conducted in the following databases: PubMed, Embase.com, Clarivate Analytics/Web of Science Core Collection, and the Wiley/Cochrane Library. The period in the databases was from inception to the 15th of October 2023. The literature search was performed by G.L.B. and M.B. The search included keywords and free text terms for (synonyms of) ‘artificial intelligence’ along with (synonyms of) ‘digestive system surgical procedures’ and ‘ultrasound.’ Using the PRESS checklist [10], this search strategy was peer-reviewed by an information specialist (G.L.B). A complete overview of search terms per database can be found in the supplementary information (see Online Appendix 1). No limitations on data were applied within this search. Studies reporting on conference proceedings, book chapters, editorials, notes, errata, letters, tombstones, or surveys were excluded from the search.

### Eligibility criteria

Studies were only considered eligible if they met the following criteria: (I) described artificial intelligence methods, (II) involved patients undergoing any type of hepatopancreatobiliary surgery, (III) involved the use of any type of ultrasound imaging (preoperative and intraoperative), (IV) clinical study. As this review is only targeting new machine learning and deep learning techniques, studies that only included statistical models without the use of artificial intelligence have been excluded from this review. Studies were also excluded if they: (I) were not written in English, (II) reported on reviews, letters, editorials, or study abstracts, (III) included children as patients. No peculiar study setting, or design was favored in the inclusion criteria.

### Study selection

Two assessors (M.B. & C.M.C.) independently conducted the title and abstract screening in accordance with the inclusion and exclusion criteria. Duplicate studies were eliminated. The remaining articles received a full-text screening by the same two assessors (M.B. & C.M.C.) to ensure compliance with the inclusion criteria. Discrepancies were solved by discussions, resulting in a consensus.

### Methodology assessment

The Probast risk of bias tool was independently applied by two assessors (M.B. & C.M.C.) to evaluate the methodological quality of included AI methods [11]. This tool evaluated the overall risk of bias based on four domains: participant selection, predictors, outcomes, and analysis.

### Data extraction

The collection of data was performed according to the Cochrane guidance for data collection, and the CHARMS checklist [12]. The following data items were extracted from each study: first author, publication year, country of research, number of patients, mean age, benefited surgical procedures, purpose of study, ultrasound modality, data type, region of interest in the data set, segmentation tool, annotation, AI subfield, internal validation, external validation, study outcomes, predictive performance (discrimination and calibration). All data items were independently extracted by two assessors (M.B. & C.M.C.). Conflicts were solved by consensus between the two assessors.

### Data synthesis

Descriptive summaries were used to illustrate applied AI subfields, benefited surgical outcomes, risk of bias assessment, model development, model performance, and validation. The discriminative abilities of AI models were presented as the mean accuracy (ACC) or area under the curve (AUC).

## Results

**Fig. 1:**
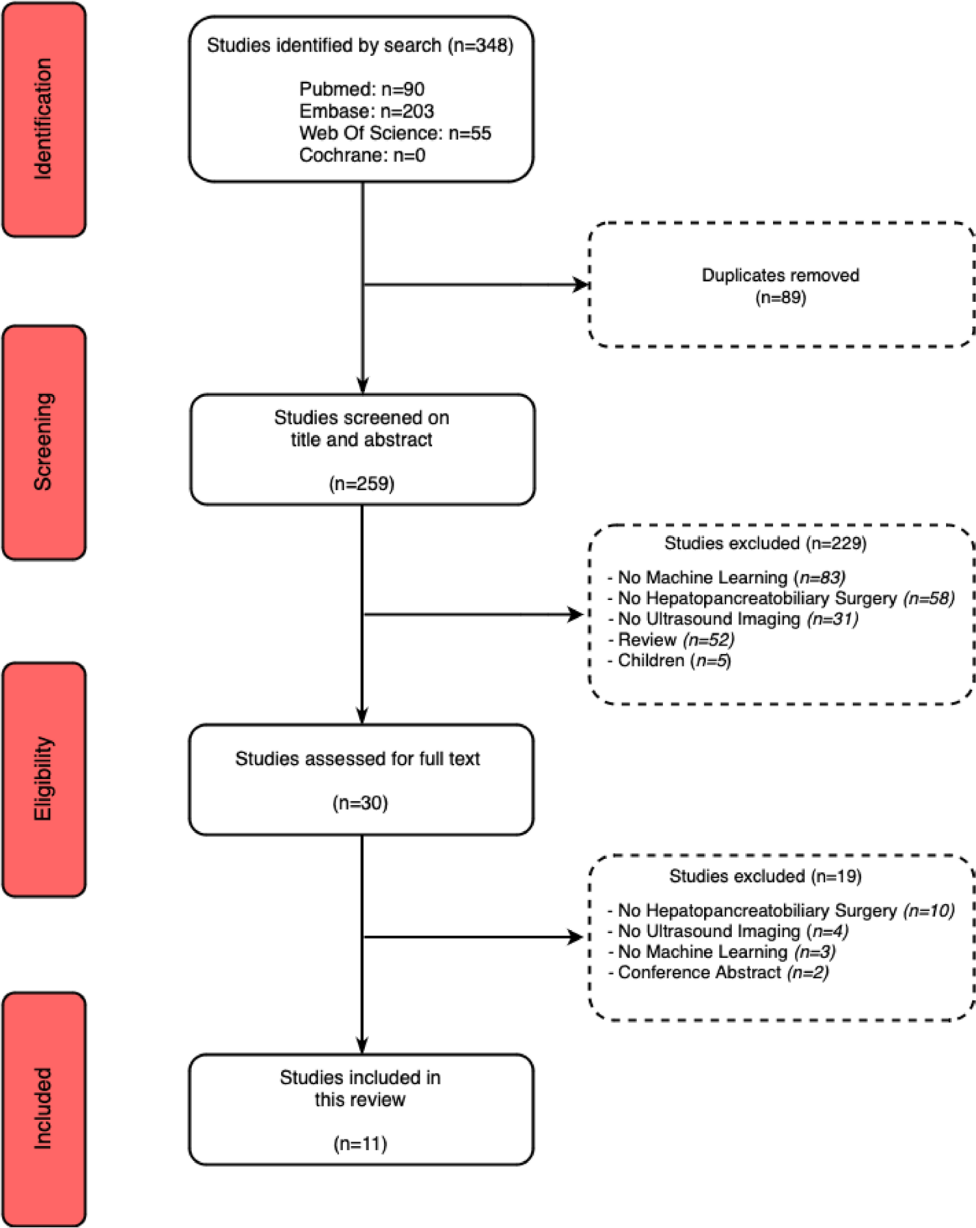
PRISMA flow chart of the search strategy and study selection

The results of the search and screening process are presented in the PRISMA flowchart (Fig. 1). Of the identified 348 records, 89 were filtered for duplicates. Subsequently, 259 records were screened on title and abstract using the Rayyan platform [13], where 229 records were excluded. After performing full-text screening on the remaining 30 records based on the eligibility criteria, 11 were included in the review.

### Surgical outcomes

The three main areas of hepatopancreatobiliary surgery benefiting from ultrasound-based AI methods were liver cancer (*n* = 6), pancreatic cancer (*n* = 4), and biliary cancer (*n* = 1). Additionally, three major categories of surgical outcomes were reported: prediction of prognosis; differentiation between two or more classes of tumoral tissue; identification of lesions within the field of view during ultrasound imaging (Table 1).

**Table 1:** General characteristics of included studies

Authors	Year	Country	Patients	Age (mean)	Benefited surgical procedures	Purpose of study	Ultra- sound modality	Data type (format)	Region of interest in training set	Segmenta- tion tool or preprocessing methods	Labeling	Feature extraction tool + AI model	AI problem type	IV & Test	EV	Predictive Performance (ACC/ AUC)
Dong et al.	2020	China	322	58	Prognosis after hepatectomy	Predict recurrence of HCC using MVI	US	Image (format not reported)	Tumoral of lesion	*MITK*	Post- treatment report	*Pyradiomics* + RF	Classification	IV	No	0.81 AUC
Huang et al.	2021	China	215	54	Prognosis after hepatectomy	Predict recurrence of HCC	CEUS	Image and video (DICOM)	Tumoral and peritumoral lesion	*ITK-SNAP*	Post- treatment report	*Ultrasomics* + LR	Classification	IV & Test	No	0.85 AUC
Mao et al.	2021	China	114	59	Diagnosis before hepatectomy	Differentiate between primary & metastatic cancer	US	Image (DICOM)	Tumoral lesion	*ITK-SNAP*	Post- treatment report	*Pyradiomics* + LR, RF, KNN, MLP; SVM	Classification	IV	No	0.77–0.82 AUC
Dong et al.	2022	China	100	59	Prognosis after hepatectomy	Predict recurrence of HCC using MVI	Kupffer phase CEUS	Image (DICOM)	Tumoral and peritumoral lesion	*3D Slicer*	Post- treatment report	*Pyradiomics* + SVM	Classification	IV	No	0.80 AUC
Zhang et al.	2022	China	172	51	Prognosis after hepatectomy	Predict recurrence of HCC	CEUS	Image (format not reported)	Tumoral and peritumoral lesion	*MaZda* & data aug- menta- tion	Post- treatment report	*MaZda* + *Resnet-50* + CNN	Classification	IV & Test	No	0.83 AUC
Barash et al.	2022	Israel	16	57	Intraoperative diagnosis for hepatectomy	Differentiate between normal tissues and focal liver lesions	IOUS	Image (format not reported)	Full image	Down- sampling	Labeling image by a radiologist	*FishNet-150* + CNN	Classification	IV & Test	No	0.80 AUC
Norton et al.	2001	USA	35	-	Diagnosis before pancreatico- duodenectomy	Differentiate between pancreatitis and pancreatic cancer	EUS	Scanned radiograph (JPEG)	Tumoral lesion	Cropping	Post- treatment report	*SOM *[28] + Neural Networks	Classification	IV	No	80% ACC
Kuwahara et al.	2019	Japan	50	70	Diagnosis before pancreatectomy	Predict malignancy of IPMNs	EUS	Image (JPEG)	Full image	Cropping	Post- treatment report	*Resnet-50* + CNN	Classification	IV	No	0.98 AUC
Tong et al.	2022	China	558	63	Diagnosis before pancreatectomy	Differentiate between PDAC and chronic pancreatitis	CEUS	Image and video (DICOM)	Tumoral and peritumoral lesion	*Labelme* & resizing	Labeling image by radiologists	*Resnet-50* + CNN	Classification	IV	Yes	0.95 AUC
Shulz et al.	2022	Germany	27	71	Diagnosis before pancreatectomy	Differentiate between malignant and benign IPMNs	EUS	Image (JPG)	Full image	Resizing & data augmentation	Post- treatment report	*EfficientNet* + CNN	Classification	IV & Test	Yes	99.6% ACC
Wang et al.	2022	China	536	55	Diagnosis before cholecystectomy	Differentiate between benign and malignant gallbladder lesions	CEUS	DICOM	Tumoral and peritumoral lesion	ITK-SNAP	Labeling image by radiologists	*Ultrasomics* + *Gradient boosting*		IV & Test	Yes	0.91 AUC

*IV* internal validation, Test internal testing, *EV* external validation, *HCC* hepatocellular carcinoma, *MVI* microvascular invasion, *ACC* accuracy, *AUC* area under the curve, *US* ultrasound, *CEUS* contrast-enhanced ultrasound, *EUS* endoscopic ultrasound, *IOUS* intraoperative ultrasound, *KNN* k-nearest neighbor, *LR* logistic regression, *MLP* multilayer perceptron, *RF* random forest, *SVM* support vector machine

Out of the six studies involving liver malignancies, AI models were used for predicting early recurrence of HCC (*n* = 4), differentiating between liver cancer and metastatic cancer (*n* = 1), and detecting focal liver lesions (*n* = 1). In four studies concerning pancreatic cancer, one AI model was developed to differentiate between benign and malignant intraductal papillary mucinous neoplasms (IPMN), while another algorithm was used to differentiate between pancreatitis and pancreatic cancer. Furthermore, one AI model aimed to predict malignant IPMNs, and one model was designed to identify pancreatic ductal adenocarcinomas versus chronic pancreatitis regions. Finally, one AI model was developed to differentiate between malignant and benign lesions of the gallbladder.

### Model development

**Fig. 2:**
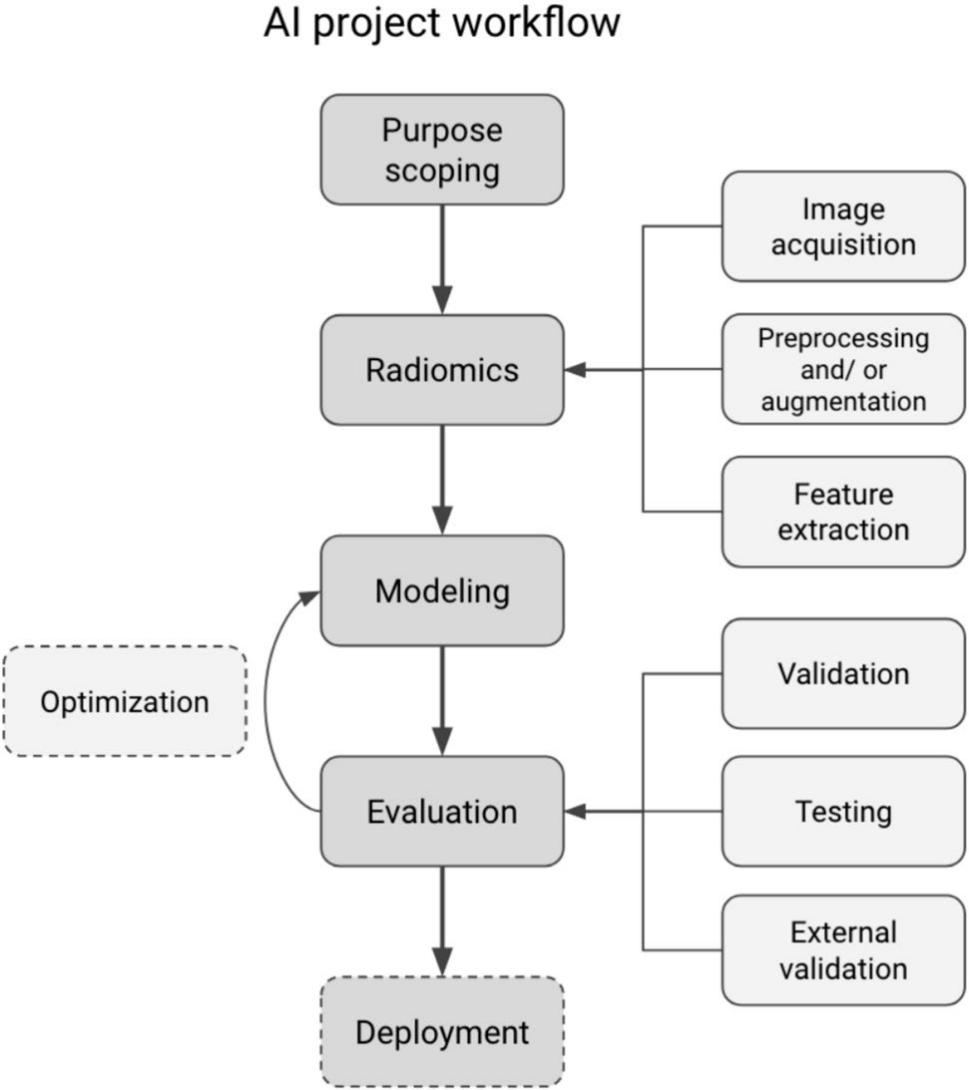
Common workflow of AI models observed within the included studies

Within included studies, the following trend was observed in the process of model development: purpose scoping, data handling, AI modeling, and evaluation. A framework depicted in Fig. 2 summarizes the applied workflows within included studies. A nomenclature of AI processes is shown in Table 2.

**Table 2:** Nomenclature of AI processes

Machine learning models	Algorithms able to process input data, train on this data to acquire a certain task, until an optimal accuracy is achieved. Examples include Random Forest, Decision Tree, SVM, Gradient Boosting models
Radiomics	Algorithms able to extract quantitative features of images that represent specific components of pathologies
Neural Networks	Algorithms able to process input data through multiple layers for pattern detection, in which the model itself assigns weights for the features within the data
Image Acquisition Formats	*DICOM:* Digital Imaging and Communications in Medicine, a high-quality standard format to digitally store medical images *JPG:* Joint Photographic Experts Group, a general low-quality format to store images
Image Segmentation	Drawing a region of interest on images to extract features from the selected region or using the complete image for feature extraction
Data Labeling	Process of identifying data (images or text files) and adding informative labels to provide context for the model
Software for Feature Extraction	Software packages in which modules are stored that can be installed for feature extraction. Examples include Pyroradiomics, Ultrasomics, Mazda, ResNet-50, EfficientNet B5, FishNet-150, IFoundry
Internal Validation Methods	*Cross-validation*: Dividing datasets into multiple subsets to use one subset for validation and the remaining subsets as training subsets. This process is repeated to validate the prediction on multiple subsets *Random Splitting:* Randomly selecting a percentage of the dataset to use as validation sets *Bootstrapping:* Resampling datasets multiple times to enable variability in validation sets

#### Image acquisition

Four ultrasound modalities were found used in the reviewed studies: external ultrasound [14, 15], intraoperative ultrasound [16], endoscopic ultrasound [17–19], and contrast-enhanced ultrasound [20–23]. All except for two studies solely used image data in modeling. The exceptions were studies with contrast-enhanced ultrasound [20, 21], which have used both image and video data. Almost half of the studies stored the data in DICOM [14, 18, 20, 21, 24] and three studies stored the same in JPEG/JPG [17–19]. One of the studies converted the image data from the radiograph to JPEG via scanning at 300 dpi [18].

#### Radiomics

Apart from two studies that used a combination of image features and clinical factors [15–22], all other studies used image data containing a region of interest (ROI) defined as the source of predictors for the model training. Except for three studies that used full image data after size reduction steps (such as cropping [18], resizing [17, 19, 21], or down-sampling [16], the remaining studies reduced the acquired image data to the ROI via image segmentation [14, 15, 20, 22, 23]. Two studies applied data augmentation in addition to the mentioned pixel size reduction [19, 23]. The image segmentation steps were mainly performed using open-source software. In the study of Norton et al. [18], the neural network was trained on features extracted from a single image.

#### Software

Labeling of datasets for model training was performed on the ROI by medical experts in five studies, whereas the remaining studies (*n* = 8) extracted semantic labels from the patients’ post-treatment reports to label the datasets. In addition, the following software packages were used for feature extraction: Pyroradiomics (*n* = 4), Ultrasomics (*n* = 1), Mazda (*n* = 1), ResNet-50 (*n* = 2), EfficientNet B5 (*n* = 1), FishNet-150 (*n *= 1), IFoundry Software (*n* = 1).

### Model training and validation

Most studies in this review have divided the dataset into training, validation, and/or test sets, for use in training and evaluating the AI models. Convolutional neural networks were the most applied artificial neural network algorithms in this review, used with different backbones such as FishNet-150 [16], Resnet-50 [17, 21, 23], or Efficient-net [19]. Other used algorithms included Random Forest, Support Vector Machine, and Gradient Boosting. In two specific studies [14, 24], several algorithms were trained on the same training cohort for selection of the algorithm with the highest accuracy.

Validation of the models was observed in all studies in either internal validation only or a combination of internal validation, testing, and/or external validation. To gain internal validation of AI models, the following methods were used: cross-validation (*n* = 7), random splitting (*n* = 3), and bootstrapping (*n* = 1). Independent testing and external validation were performed in five and three studies, respectively.

### Model performance

The discriminative abilities of ultrasound-based AI models varied between 0.8 and 0.99. In predicting microvascular invasion of HCC, AUCs of AI models ranging between 0.8 and 0.84 were achieved. Differentiation between primary and metastatic liver cancer was performed with an AUC of 0.82. Additionally, for the intraoperative identification of focal liver lesions, an AUC of 0.8 was discovered. AI models for the differentiation between benign and malignant IPMNs have shown AUCs from 0.91 to 0.99. An AUC of 0.98 was discovered for the identification of PDAC and chronic pancreatitis. In addition, differentiation between pancreatitis and pancreatic cancer was performed with an AUC of 0.80. Differentiation between malign and benign gallbladder masses was achieved with an AUC of 0.91. Three studies extended their evaluation to compare the prediction and observation and reported it in the form of a calibration plot [21] or decision curve analysis [22, 23].

### Methodological quality assessment

All included studies including AI models were based on a retrospective study design. According to the Probast risk of bias tool for AI models, the majority of studies received a low risk of bias score for the domains ‘participants,’ ‘predictors,’ and ‘outcomes.’ For most studies, the domain of ‘analysis’ has received an unclear risk of bias score due to improper measures to adjust for missing data and overfitting. Therefore, a low overall bias was reported for 30% of the studies, whereas 30% received a high overall bias. In addition, an unclear overall bias was given for 40% of the studies (Table. 3).

**Table 3:**
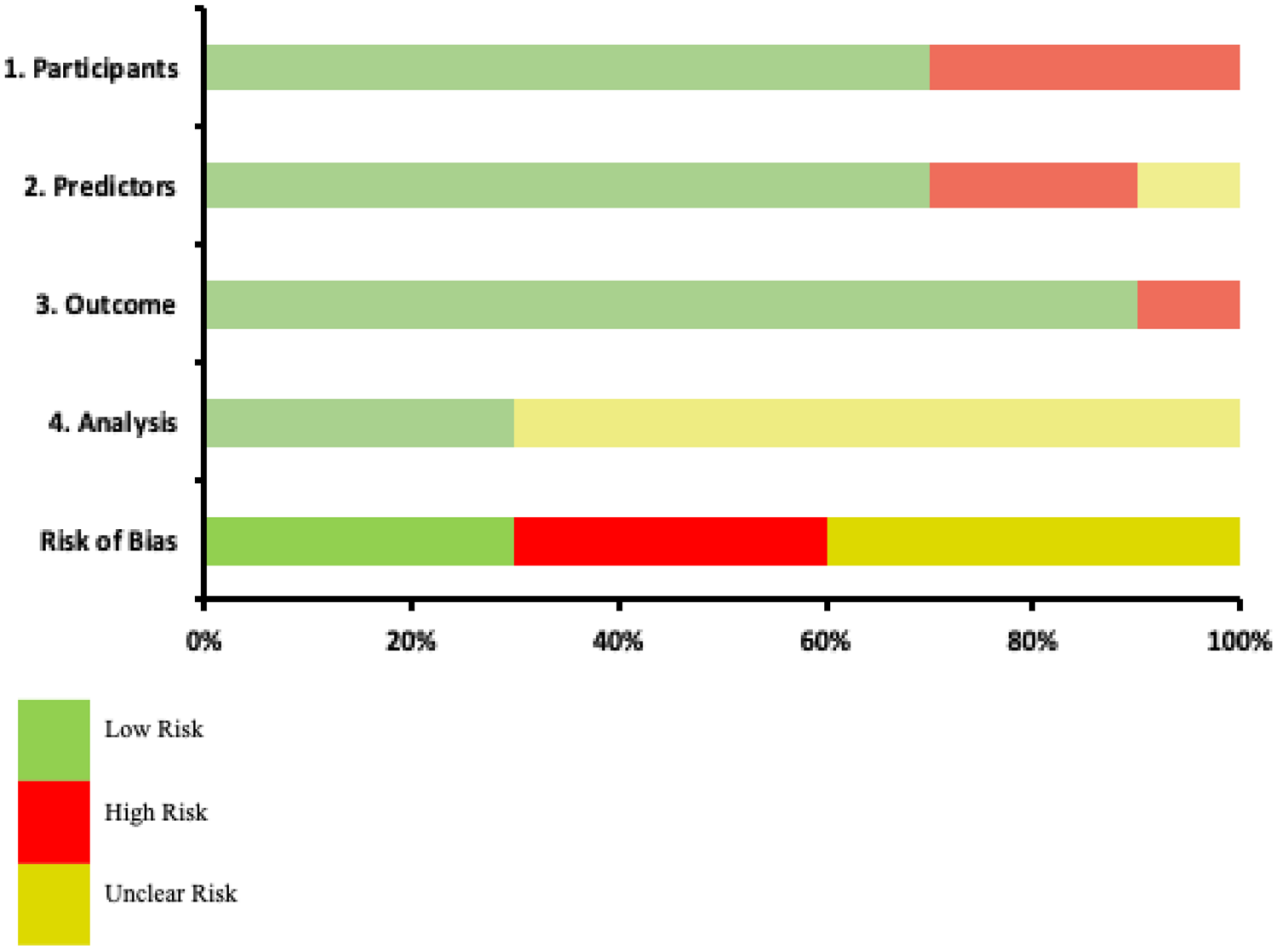
Methodological quality assessment of AI models, according to Probast risk of bias tool

## Discussion

This review addresses the current AI models on ultrasound imaging within hepatopancreatobiliary surgery. All studies used similar approaches toward AI model development, by combining radiomics and AI models toward preoperatively predicting tumor recurrence and differentiation of tumoral tissue types. These ultrasound-based AI models show promising AUCs regarding the prognosis of hepatic and pancreatic cancer, as AUCs have ranged between 0.77 and 0.99.

In clinical practice, preoperatively understanding tumoral behavior could guide surgeons in deciding between conservative or surgical treatment plans. As radiomics features are closely related to tumoral microstructures and reveal intratumoral heterogeneity, these features could be extracted to recognize tumoral behavior based on ultrasound images [25]. As an example, Dong et al. [15] have demonstrated that by using radiomics, microvascular invasion of HCC can be predicted without the need for postoperative biopsy specimens and histological examination. The ability of AI models to differentiate between various tumoral tissue has been emphasized in several studies. Differentiations have been made between primary and metastatic cancer [14], benign and malign IPMNs [19], pancreatitis and pancreatic cancer [18, 21], malign and benign gallbladder masses [24], showing AUCs of 0.82, 0.99, 0.95, and 0.91, respectively. Using this ability can prevent overtreatment and missing invasive carcinomas, therefore enhancing personalized treatment strategies. Additionally, a Convolutional neural networks model was applied on intraoperative ultrasound images to differentiate between normal liver tissue and focal lesions, showing an AUC of 0.80 [16]. As such an application is rarely reported, this study illustrates that surgeons could even be assisted intraoperatively to prevent overlooking lesions and possible metastasis during the surgical procedure. Ultimately, ultrasound-based AI models could have the largest impact in clinical practice due to their ability to differentiate accurately between benign and malignant lesions. Tumoral lesions are often found incidentally on CT scans, but this modality lacks the proper resolution to characterize specific tumoral features, especially when tumors are small scaled. With ultrasound-based AI models, morphological features could be extracted to reveal tumors in an early stage and facilitate optimal treatment plans for the patients.

As all ultrasound-based AI models demonstrated AUCs above 0.8, the discriminative ability of these models could be described as promising. This ability was emphasized by the study of Kuwahara et al. [17], in which the ultrasound-based AI model has even surpassed human diagnosis in predictive accuracy. In addition, compared to other imaging modalities, ultrasonography is easily applicable, quickly accessible, and does not cause patient radiation.

In real world settings, AI models have been implemented to detect safe dissection planes during robotic surgery, in which AI was able to provide accurate intraoperative feedback during the procedure [26]. Additionally, automatic visualization of structures such as nerves have been accomplished during laparoscopic gastrointestinal surgery [27]. Within hepatopancreatobiliary surgery, AI models have demonstrated AUCs up to 0.85 in predicting surgical outcomes such early HCC recurrence, response to chemotherapy, and postoperative complications [28–30]. AI models have shown superiority in predictive capabilities compared to conventional logistic regression models and risk calculators. However, to pre-operatively predict such surgical outcomes in daily practice, large-scale datasets are required to improve the performance of the models. In addition, multicenter studies are required to externally validate the accuracies of these models. It is vital to maintain identical workflow of the AI models, including imaging formats, data variables, feature extraction methods, and the number of patients within datasets.

In terms of clinical practicality, AI models might also encounter a few challenges. Inter- and intra-observer variability may influence the generalizability of the predictions, as the quality of ultrasound images is highly dependent on the operator. Another challenge could be the dynamicity of intraoperative ultrasound imaging. Within the operation room, ultrasound is analyzed on-site, whereas radiomics require captured and stored images to extract quantitative features.

To demonstrate the potential of ultrasound-based AI models in clinical practice, the implementation steps are essential. Using Fig. 2, which summarized the most adopted workflow in designing an AI solution within this review, the critical steps, and suggestions for the implementation of an ultrasound-based AI solution for a selected GI surgical procedure are elaborated. The first key step is defining a task type that can be solved using ultrasound image features. Common task types and their example applications are classification (e.g., categorizing lesions to tissue types), regression (e.g., staging of tumor), or clustering (automatic image segmentation of lesion). This step is frequently formulated toward imitating a current clinical procedure, with the intention of automating or standardizing the manual procedure. For example, classifying the marked lesion into appropriate tissue categories based on radiological interpretation [14, 15, 19]. Another observed approach in purpose scoping is relating postoperative data (e.g., pathology report) to preoperative ultrasound images, in order to deliver diagnoses that otherwise will not be possible following conventional clinical procedures [15, 22].

The next step involves extracting features from the ultrasound images. As image quality has an impact on the model performance [31], it is recommended to prepare the images in a lossless compression format (e.g., DICOM with JPEG 2000 compression). Preprocessing is commonly applied prior to image feature extraction, to reduce the effect of outliers and biases that could result from the acquisition of the images. In practice, there are generally two approaches in feature extraction: using a standalone software package (e.g., *Pyroradiomics, MaZda, Ultrasomics*) which outputs extracted image features to the model, or using integrated feature extraction network (commonly known as backbone) in a deep learning model. It is recommended to refer to the Image Biomarker Standardization Initiative standard [32] for designing the radiomics workflow. To avoid model overfitting, datasets should be generally divided in a training and independent testing set by using cross-validation or a random-split approach [33]. A decision curve analysis should be used to report the comparison between model performance and observed clinical risks, indicating the usability of the AI model in clinical settings [34].

The results of this review should be interpreted in consideration of several limitations. First, only studies involving hepatopancreatobiliary oncology were found with the current search strategy, this might indicate a limited extent of the search strategy, as only general terms and synonyms were used for AI, ultrasonography, and hepatopancreatobiliary surgery. Second, a comparative meta-analysis of AI models was not achievable, due to heterogeneous methodologies of the AI studies. Third, as most AI models were based on retrospective studies, none of the models were implemented in clinical practice. Therefore, the accuracies of the AI models should be considered as performances specific to the respective reported studies.

Future studies should focus on new task types, external validation, and reproducibility for the developed AI models. Classification was the major task type in all the articles, in which the models were trained to classify the new input US images into one of the trained classes. Other task types of AI including regression and clustering could be explored to train models that predict continuous annotation or automatic image segmentation. For instance, ultrasound as both a qualitative and quantitative imaging modality [35] could provide tissue mechanical properties data (e.g., elastography) in addition to the spatial image data, for staging of tumor grade [36] and automatic identification of tumor boundary on the ultrasound images.

As external validation was missing in most studies, independent datasets should be used for validating the usability, performance, and risks of the AI model. External validation is essential to support the generalizability of AI models, and eventually facilitate the clinical implementation [37]. Before clinical implementation can occur, large datasets need to become available to enable external validation and improve the predictive accuracies of ultrasound-based AI models. In addition, the development of AI models is usually not properly illustrated, resulting in a lack of reproducibility and transparency. Collective initiatives, such as the FAIR (Findability, Accessibility, Interoperability, Reusability) principle and guideline [38], could help in overcoming the problem [39]. These guidelines could help authors by providing a general checklist on how to properly secure reproducibility, transparency, and reusability for the AI model. Prospective studies, using large datasets of several hospitals, should be established to evaluate the definitive validation and predictive performance of ultrasound-based AI models within hepatopancreatobiliary surgery.

## Conclusion

In conclusion, this review illustrates promising performances of AI models in predicting early tumoral recurrence and differentiating between tumoral tissue types for patients undergoing hepatopancreatobiliary surgery. Despite the promising accuracies in this review, the current literature only reported early-phase applications of ultrasound-based AI models, involving mostly retrospective studies without external validation. Whether these results will remain valid during prospective studies is yet to be determined.

### Supplementary Information

Below is the link to the electronic supplementary material.

Supplementary file1 (DOCX 18 kb)

## References

[1] Machi J, Sigel B, Zaren HA, Kurohiji T, Yamashita Y (1993) Operative ultrasonography during hepatobiliary and pancreatic surgery. World J Surg 17(5):640–646 8273386 10.1007/BF01659130

[2] Montoya J, Stawicki SP, Evans DC, Bahner DP, Sparks S, Sharpe RP et al (2016) From FAST to E-FAST: an overview of the evolution of ultrasound-based traumatic injury assessment. Eur J Trauma Emerg Surg 42(2):119–126 26038031 10.1007/s00068-015-0512-1

[3] Sooklal S, Chahal P (2020) Endoscopic ultrasound. Surg Clin North Am 100(6):1133–1150 33128884 10.1016/j.suc.2020.07.003

[4] Smereczyński A, Kołaczyk K (2018) Pitfalls in ultrasound imaging of the stomach and the intestines. J Ultrason 18(74):207–211 30451403 10.15557/JoU.2018.0031 PMC6442211

[5] Choe J, Wortman JR, Michaels A, Sarma A, Fulwadhva UP, Sodickson AD (2019) Beyond appendicitis: ultrasound findings of acute bowel pathology. Emerg Radiol 26(3):307–317 30661212 10.1007/s10140-019-01670-7

[6] Tomizawa M, Shinozaki F, Hasegawa R, Shirai Y, Motoyoshi Y, Sugiyama T et al (2017) Abdominal ultrasonography for patients with abdominal pain as a first-line diagnostic imaging modality. Exp Ther Med 13(5):1932–1936 28565789 10.3892/etm.2017.4209 PMC5443284

[7] Lee ES, Lee JM (2014) Imaging diagnosis of pancreatic cancer: a state-of-the-art review. World J Gastroenterol 20(24):7864–7877 24976723 10.3748/wjg.v20.i24.7864 PMC4069314

[8] Shur JD, Doran SJ, Kumar S, Dafydd DA, Downey K, O’Connor JPB et al (2021) Radiomics in oncology: a practical guide. Radiographics 41(6):1717–1732 34597235 10.1148/rg.2021210037 PMC8501897

[9] Visvikis D, Cheze Le Rest C, Jaouen V, Hatt M (2019) Artificial intelligence, machine (deep) learning and radio(geno)mics: definitions and nuclear medicine imaging applications. Eur J Nucl Med Mol Imaging 46(13):2630–2637 31280350 10.1007/s00259-019-04373-w

[10] McGowan J, Sampson M, Salzwedel DM, Cogo E, Foerster V, Lefebvre C (2016) PRESS peer review of electronic search strategies: 2015 guideline statement. J Clin Epidemiol 75:40–46 27005575 10.1016/j.jclinepi.2016.01.021

[11] Wolff RF, Moons KGM, Riley RD, Whiting PF, Westwood M, Collins GS et al (2019) PROBAST: a tool to assess the risk of bias and applicability of prediction model studies. Ann Intern Med 170(1):51–58 30596875 10.7326/M18-1376

[12] Moons KG, de Groot JA, Bouwmeester W, Vergouwe Y, Mallett S, Altman DG et al (2014) Critical appraisal and data extraction for systematic reviews of prediction modelling studies: the CHARMS checklist. PLoS Med 11(10):e1001744 25314315 10.1371/journal.pmed.1001744 PMC4196729

[13] Ouzzani M, Hammady H, Fedorowicz Z, Elmagarmid A (2016) Rayyan-a web and mobile app for systematic reviews. Syst Rev 5(1):210 27919275 10.1186/s13643-016-0384-4 PMC5139140

[14] Mao B, Ma J, Duan S, Xia Y, Tao Y, Zhang L (2021) Preoperative classification of primary and metastatic liver cancer via machine learning-based ultrasound radiomics. Eur Radiol 31(7):4576–4586 33447862 10.1007/s00330-020-07562-6

[15] Dong Y, Zhou L, Xia W, Zhao XY, Zhang Q, Jian JM et al (2020) Preoperative prediction of microvascular invasion in hepatocellular carcinoma: initial application of a radiomic algorithm based on grayscale ultrasound images. Front Oncol 10:353 32266138 10.3389/fonc.2020.00353 PMC7096379

[16] Barash Y, Klang E, Lux A, Konen E, Horesh N, Pery R et al (2022) Artificial intelligence for identification of focal lesions in intraoperative liver ultrasonography. Langenbecks Arch Surg 407(8):3553–3560 36068378 10.1007/s00423-022-02674-7

[17] Kuwahara T, Hara K, Mizuno N, Okuno N, Matsumoto S, Obata M et al (2019) Usefulness of deep learning analysis for the diagnosis of malignancy in intraductal papillary mucinous neoplasms of the pancreas. Clin Transl Gastroenterol 10(5):1–8 31117111 10.14309/ctg.0000000000000045 PMC6602761

[18] Norton ID, Zheng Y, Wiersema MS, Greenleaf J, Clain JE, Dimagno EP (2001) Neural network analysis of EUS images to differentiate between pancreatic malignancy and pancreatitis. Gastrointest Endosc 54(5):625–629 11677484 10.1067/mge.2001.118644

[19] Schulz D, Heilmaier M, Phillip V, Treiber M, Mayr U, Lahmer T et al (2023) Accurate prediction of histological grading of intraductal papillary mucinous neoplasia using deep learning. Endoscopy 55(5):415–422 36323331 10.1055/a-1971-1274

[20] Huang H, Ruan SM, Xian MF, Li MD, Cheng MQ, Li W et al (2022) Contrast-enhanced ultrasound-based ultrasomics score: a potential biomarker for predicting early recurrence of hepatocellular carcinoma after resection or ablation. Br J Radiol 95(1130):20210748 34797687 10.1259/bjr.20210748 PMC8822579

[21] Tong T, Gu J, Xu D, Song L, Zhao Q, Cheng F et al (2022) Deep learning radiomics based on contrast-enhanced ultrasound images for assisted diagnosis of pancreatic ductal adenocarcinoma and chronic pancreatitis. BMC Med 20(1):74 35232446 10.1186/s12916-022-02258-8 PMC8889703

[22] Dong Y, Zuo D, Qiu YJ, Cao JY, Wang HZ, Yu LY et al (2022) Preoperative prediction of microvascular invasion (MVI) in hepatocellular carcinoma based on kupffer phase radiomics features of sonazoid contrast-enhanced ultrasound (SCEUS): a prospective study. Clin Hemorheol Microcirc 81(1):97–107 35001883 10.3233/CH-211363

[23] Zhang H, Huo F (2022) Prediction of early recurrence of HCC after hepatectomy by contrast-enhanced ultrasound-based deep learning radiomics. Front Oncol 12:930458 36248986 10.3389/fonc.2022.930458 PMC9554932

[24] Wang LF, Wang Q, Mao F, Xu SH, Sun LP, Wu TF et al (2023) Risk stratification of gallbladder masses by machine learning-based ultrasound radiomics models: a prospective and multi-institutional study. Eur Radiol 33(12):8899–8911 37470825 10.1007/s00330-023-09891-8

[25] Guo Y, Hu Y, Qiao M, Wang Y, Yu J, Li J et al (2018) Radiomics analysis on ultrasound for prediction of biologic behavior in breast invasive ductal carcinoma. Clin Breast Cancer 18(3):e335–e344 28890183 10.1016/j.clbc.2017.08.002

[26] Knudsen JE, Ghaffar U, Ma R, Hung AJ (2024) Clinical applications of artificial intelligence in robotic surgery. J Robot Surg 18(1):102 38427094 10.1007/s11701-024-01867-0 PMC10907451

[27] Ryu S, Goto K, Imaizumi Y, Nakabayashi Y (2024) Laparoscopic colorectal surgery with anatomical recognition with artificial intelligence assistance for nerves and dissection layers. Ann Surg Oncol 31(3):1690–1691 38017127 10.1245/s10434-023-14633-7

[28] Qiao G, Li J, Huang A, Yan Z, Lau WY, Shen F (2014) Artificial neural networking model for the prediction of post-hepatectomy survival of patients with early hepatocellular carcinoma. J Gastroenterol Hepatol 29(12):2014–2020 24989634 10.1111/jgh.12672

[29] Zhu HB, Xu D, Ye M, Sun L, Zhang XY, Li XT et al (2021) Deep learning-assisted magnetic resonance imaging prediction of tumor response to chemotherapy in patients with colorectal liver metastases. Int J Cancer 148(7):1717–1730 33284998 10.1002/ijc.33427

[30] Merath K, Hyer JM, Mehta R, Farooq A, Bagante F, Sahara K et al (2020) Use of machine learning for prediction of patient risk of postoperative complications after liver, pancreatic, and colorectal surgery. J Gastrointest Surg 24(8):1843–1851 31385172 10.1007/s11605-019-04338-2

[31] Jonske F, Dederichs M, Kim MS, Keyl J, Egger J, Umutlu L et al (2022) Deep learning-driven classification of external DICOM studies for PACS archiving. Eur Radiol 32(12):8769–8776 35788757 10.1007/s00330-022-08926-w PMC9705446

[32] Zwanenburg A, Vallières M, Abdalah MA, Aerts HJWL, Andrearczyk V, Apte A et al (2020) The image biomarker standardization initiative: standardized quantitative radiomics for high-throughput image-based phenotyping. Radiology 295(2):328–338 32154773 10.1148/radiol.2020191145 PMC7193906

[33] Hutter F, Kotthoff L, Vanschoren J (2019) Automated machine learning: methods, systems, challenges. In: Hutter F, Kotthoff L, Vanschoren J (eds) The springer series on challenges in machine learning. Springer, Berlin, pp 113–134

[34] Vickers AJ, Holland F (2021) Decision curve analysis to evaluate the clinical benefit of prediction models. Spine J 21(10):1643–1648 33676020 10.1016/j.spinee.2021.02.024 PMC8413398

[35] Oelze ML, Mamou J (2016) Review of quantitative ultrasound: envelope statistics and backscatter coefficient imaging and contributions to diagnostic ultrasound. IEEE Trans Ultrason Ferroelectr Freq Control 63(2):336–351 26761606 10.1109/TUFFC.2015.2513958 PMC5551399

[36] Valero M, Robles-Medranda C (2017) Endoscopic ultrasound in oncology: an update of clinical applications in the gastrointestinal tract. World J Gastrointest Endosc 9(6):243–254 28690767 10.4253/wjge.v9.i6.243 PMC5483416

[37] Kim DW, Jang HY, Kim KW, Shin Y, Park SH (2019) Design characteristics of studies reporting the performance of artificial intelligence algorithms for diagnostic analysis of medical images: results from recently published papers. Korean J Radiol 20(3):405–410 30799571 10.3348/kjr.2019.0025 PMC6389801

[38] Wilkinson MD, Dumontier M, Aalbersberg IJ, Appleton G, Axton M, Baak A et al (2016) The FAIR guiding principles for scientific data management and stewardship. Sci Data 3:160018 26978244 10.1038/sdata.2016.18 PMC4792175

[39] Lambin P, Leijenaar RTH, Deist TM, Peerlings J, de Jong EEC, van Timmeren J et al (2017) Radiomics: the bridge between medical imaging and personalized medicine. Nat Rev Clin Oncol 14(12):749–762 28975929 10.1038/nrclinonc.2017.141

