# Supplementary material for: Artificial intelligence-aided ultrasound imaging in hepatopancreatobiliary surgery: where are we now?
Source: Surg Endosc. 2024 Aug 19;38(9):4869–79. doi: 10.1007/s00464-024-11130-0 (PMC11362182; doi:10.1007/s00464-024-11130-0)
Supplement: Supplementary file 1 — Supplementary file1 (DOCX 18 kb) [file 464_2024_11130_MOESM1_ESM.docx]

**Appendix 1**

# Final Search 15/10/2023

## PubMed (90)

| **Search** | **Query** | **Results** |
| --- | --- | --- |
| #4 | Search: **#1 AND #2 AND #3** Sort by: **Most Recent** | [9](https://pubmed.ncbi.nlm.nih.gov/?term=%231+AND+%232+AND+%233&sort=date&ac=no)0 |
| #3 | Search: **"Ultrasonography"[Mesh] OR ultrasound*[tiab] OR "ultra sound*"[tiab] OR Echotomograph*[tiab] OR Echo-tomograph*[tiab] OR Echotomogram*[tiab] OR Echo-tomogram*[tiab] OR Echograph*[tiab] OR Echo-graph*[tiab] OR Echogram*[tiab] OR Echo-gram*[tiab] OR Sonograph*[tiab] OR Sono-graph*[tiab] OR Sonogram*[tiab] OR Sono-gram*[tiab] OR Ultrasonograph*[tiab] OR Ultra-sonograph*[tiab] OR Ultrasonic*[tiab] OR Ultra-sonic*[tiab] OR Ultrasonogram*[tiab] OR Ultra-sonogram*[tiab] OR Echoscop*[tiab] OR Echo-scop*[tiab]** Sort by: **Most Recent** | [754,](https://pubmed.ncbi.nlm.nih.gov/?term=%22Ultrasonography%22%5BMesh%5D+OR%0Aultrasound%2A%5Btiab%5D+OR%0A%E2%80%9Cultra+sound%2A%E2%80%9D%5Btiab%5D+OR+%0A%0AEchotomograph%2A%5Btiab%5D+OR%0AEcho-tomograph%2A%5Btiab%5D+OR%0AEchotomogram%2A%5Btiab%5D+OR%0AEcho-tomogram%2A%5Btiab%5D+OR%0A%0A%0AEchograph%2A%5Btiab%5D+OR%0AEcho-graph%2A%5Btiab%5D+OR%0AEchogram%2A%5Btiab%5D+OR%0AEcho-gram%2A%5Btiab%5D+OR%0A%0ASonograph%2A%5Btiab%5D+OR%0ASono-graph%2A%5Btiab%5D+OR%0ASonogram%2A%5Btiab%5D+OR%0ASono-gram%2A%5Btiab%5D+OR%0A%0AUltrasonograph%2A%5Btiab%5D+OR+%0AUltra-sonograph%2A%5Btiab%5D+OR%0AUltrasonic%2A%5Btiab%5D+OR%0AUltra-sonic%2A%5Btiab%5D+OR%0AUltrasonogram%2A%5Btiab%5D+OR%0AUltra-sonogram%2A%5Btiab%5D+OR%0A%0AEchoscop%2A%5Btiab%5D+OR%0AEcho-scop%2A%5Btiab%5D&sort=date&ac=no)254 |
| #2 | Search: **"Digestive System Surgical Procedures"[Mesh] OR "Bariatric Surgery"[Mesh] OR "Laparotomy"[Mesh] OR "Roux-en-Y"[Tiab] OR "Cholecystostom*"[Tiab] OR "Choledochostom*"[Tiab] OR "Gastroenterostom*"[Tiab] OR "Jejunoileal Bypass*"[Tiab] OR "Pancreaticojejunostom*"[Tiab] OR "Peritoneovenous Shunt"[Tiab] OR "Portoenterostom*"[Tiab] OR "Gastric Bypass*"[Tiab] OR "Appendectom*"[Tiab] OR "Cholecystectom*"[Tiab] OR "Sphincterotom*"[Tiab] OR "Colectom*"[Tiab] OR "Cecostom*"[Tiab] OR "Colostom*"[Tiab] OR "Duodenostom*"[Tiab] OR "Ileostom*"[Tiab] OR "Jejunostom*"[Tiab] OR "Esophagectom*"[Tiab] OR "Hemorrhoidectom*"[Tiab] OR "Hepatectom*"[Tiab] OR "Liver Transplant*"[Tiab] OR "Pancreas Transplant*"[Tiab] OR "Pancreatectom*"[Tiab] OR "Pancreaticoduodenectom*"[Tiab] OR "Proctectom*"[Tiab] OR "gastrectom*"[tiab] OR "Gastrostom*"[tiab] OR "Esophagoplast*"[tiab] OR "Esophagostom*"[tiab] OR "Hepatectom*"[tiab]** Sort by: **Most Recent** | [540,](https://pubmed.ncbi.nlm.nih.gov/?term=%22Digestive+System+Surgical+Procedures%22%5BMesh%5D+OR%0A%22Bariatric+Surgery%22%5BMesh%5D+OR%0A%22Laparotomy%22%5BMesh%5D+OR%0A%22Roux-en-Y%22%5BTiab%5D+OR%0A%22Cholecystostom%2A%22%5BTiab%5D+OR%0A%22Choledochostom%2A%22%5BTiab%5D+OR%0A%22Gastroenterostom%2A%22%5BTiab%5D+OR%0A%22Jejunoileal+Bypass%2A%22%5BTiab%5D+OR%0A%22Pancreaticojejunostom%2A%22%5BTiab%5D+OR%0A%22Peritoneovenous+Shunt%22%5BTiab%5D+OR%0A%22Portoenterostom%2A%22%5BTiab%5D+OR%0A%22Gastric+Bypass%2A%22%5BTiab%5D+OR%0A%22Appendectom%2A%22%5BTiab%5D+OR%0A%22Cholecystectom%2A%22%5BTiab%5D+OR%0A%22Sphincterotom%2A%22%5BTiab%5D+OR%0A%22Colectom%2A%22%5BTiab%5D+OR%0A%22Cecostom%2A%22%5BTiab%5D+OR%0A%22Colostom%2A%22%5BTiab%5D+OR%0A%22Duodenostom%2A%22%5BTiab%5D+OR%0A%22Ileostom%2A%22%5BTiab%5D+OR%0A%22Jejunostom%2A%22%5BTiab%5D+OR%0A%22Esophagectom%2A%22%5BTiab%5D+OR%0A%22Hemorrhoidectom%2A%22%5BTiab%5D+OR%0A%22Hepatectom%2A%22%5BTiab%5D+OR%0A%22Liver+Transplant%2A%22%5BTiab%5D+OR%0A%22Pancreas+Transplant%2A%22%5BTiab%5D+OR%0A%22Pancreatectom%2A%22%5BTiab%5D+OR%0A%22Pancreaticoduodenectom%2A%22%5BTiab%5D+OR%0A%22Proctectom%2A%22%5BTiab%5D+OR%0A%E2%80%9Cgastrectom%2A%E2%80%9D%5Btiab%5D+OR%0A%E2%80%9CGastrostom%2A%E2%80%9D%5Btiab%5D+OR%0A%E2%80%9CEsophagoplast%2A%E2%80%9D%5Btiab%5D+OR%0A%E2%80%9CEsophagostom%2A%E2%80%9D%5Btiab%5D+OR%0A%E2%80%9CHepatectom%2A%E2%80%9D%5Btiab%5D&sort=date&ac=no)552 |
| #1 | Search: **"Machine Learning"[Mesh] OR "Machine Learning"[tiab] OR "machine intelligen*"[tiab] OR "machine vision*"[tiab] OR "machine learning"[tiab] OR "transfer learning"[tiab] OR "deep learning"[tiab] OR "neural network*"[tiab] OR "support vector machine*"[tiab] OR "automatic segmentation*"[tiab] OR "Long short term memory"[tiab] OR "LSTM"[tiab] OR "supervised learning"[tiab] OR "unsupervised learning"[tiab] OR "reinforcement learning*"[tiab] OR "hierarchical learning*" [tiab] OR "Image Interpretation*"[tiab] OR "Prediction model*"[tiab] OR "image recognition"[tiab] OR "perceptron"[tiab]** Sort by: **Most Recent** | [274,](https://pubmed.ncbi.nlm.nih.gov/?term=%22Machine+Learning%22%5BMesh%5D+OR%0A%22Machine+Learning%22%5Btiab%5D+OR%0A%E2%80%9Cmachine+intelligen%2A%E2%80%9D%5Btiab%5D+OR%0A%E2%80%9Cmachine+vision%2A%E2%80%9D%5Btiab%5D+OR%0A%E2%80%9Cmachine+learning%E2%80%9D%5Btiab%5D+OR%0A%E2%80%9Ctransfer+learning%E2%80%9D%5Btiab%5D+OR%0A%E2%80%9Cdeep+learning%E2%80%9D%5Btiab%5D+OR%0A%E2%80%9Cneural+network%2A%E2%80%9D%5Btiab%5D+OR%0A%E2%80%9Csupport+vector+machine%2A%E2%80%9D%5Btiab%5D+OR%0A%E2%80%9Cautomatic+segmentation%2A%E2%80%9D%5Btiab%5D+OR+%0A%E2%80%9CLong+short+term+memory%E2%80%9D%5Btiab%5D+OR%0A%E2%80%9CLSTM%E2%80%9D%5Btiab%5D+OR%0A%E2%80%9Csupervised+learning%E2%80%9D%5Btiab%5D+OR%0A%E2%80%9Cunsupervised+learning%E2%80%9D%5Btiab%5D+OR%0A%E2%80%9Creinforcement+learning%2A%E2%80%9D%5Btiab%5D+OR%0A%E2%80%9Chierarchical+learning%2A%E2%80%9D+%5Btiab%5D+OR%0A%E2%80%9CImage+Interpretation%2A%E2%80%9D%5Btiab%5D+OR%0A%E2%80%9CPrediction+model%2A%E2%80%9D%5Btiab%5D+OR%0A%E2%80%9Cimage+recognition%E2%80%9D%5Btiab%5D+OR%0A%E2%80%9Cperceptron%E2%80%9D%5Btiab%5D&sort=date&ac=no)342 |

## Embase (203)

| **Search** | **Query** | **Results** |
| --- | --- | --- |
| #4 | **#1 AND #2 AND #3** | 203 |
| #3 | 'echography'/exp OR (ultrasound* OR ultra-sound* OR Echotomograph* OR Echo-tomograph* OR Echotomogram* OR Echo-tomogram* OR Echograph* OR Echo-graph* OR Echogram* OR Echo-gram* OR Sonograph* OR Sono-graph* OR Sonogram* OR Sono-gram* OR Ultrasonograph* OR Ultra-sonograph* OR Ultrasonic* OR Ultra-sonic* OR Ultrasonogram* OR Ultra-sonogram* OR Echoscop* OR Echo-scop*):ti,ab,kw | 1,402,801 |
| #2 | 'gastrointestinal surgery'/exp OR 'laparotomy'/exp OR 'biliary tract surgery'/exp OR (‘Roux-en-Y’ OR ‘Cholecystostom*’ OR ‘Choledochostom*’ OR ‘Gastroenterostom*’ OR ‘Jejunoileal Bypass*’ OR ‘Pancreaticojejunostom*’ OR ‘Peritoneovenous Shunt’ OR ‘Portoenterostom*’ OR ‘Gastric Bypass*’ OR ‘Appendectom*’ OR ‘Cholecystectom*’ OR ‘Sphincterotom*’ OR ‘Colectom*’ OR ‘Cecostom*’ OR ‘Colostom*’ OR ‘Duodenostom*’ OR ‘Ileostom*’ OR ‘Jejunostom*’ OR ‘Esophagectom*’ OR ‘Hemorrhoidectom*’ OR ‘Hepatectom*’ OR ‘Liver Transplant*’ OR ‘Pancreas Transplant*’ OR ‘Pancreatectom*’ OR ‘Pancreaticoduodenectom*’ OR ‘Proctectom*’ OR ‘gastrectom*’ OR ‘Gastrostom*’ OR ‘Esophagoplast*’ OR ‘Esophagostom*’ OR ‘Hepatectom*’):ti,ab,kw | 828,667 |
| #1 | 'machine learning'/exp OR (‘Machine Learning’ OR ‘machine intelligen*’ OR ‘machine vision*’ OR ‘machine learning’ OR ‘transfer learning’ OR ‘deep learning’ OR ‘neural network*’ OR ‘support vector machine*’ OR ‘automatic segmentation*’ OR ‘Long short term memory’ OR ‘LSTM’ OR ‘supervised learning’ OR ‘unsupervised learning’ OR ‘reinforcement learning*’ OR ‘hierarchical learning*’ OR ‘Image Interpretation*’ OR ‘Prediction model*’ OR ‘image recognition’ OR ‘perceptron’):ti,ab,kw | 544,836 |

## Web of Science (55)

| **Search** | **Query** | **Results** |
| --- | --- | --- |
| #4 | **#1 AND #2 AND #3** | 55 |
| #3 | TS=(ultrasound* OR ultra-sound* OR Echotomograph* OR Echo-tomograph* OR Echotomogram* OR Echo-tomogram* OR Echograph* OR Echo-graph* OR Echogram* OR Echo-gram* OR Sonograph* OR Sono-graph* OR Sonogram* OR Sono-gram* OR Ultrasonograph* OR  Ultra-sonograph* OR Ultrasonic* OR Ultra-sonic* OR Ultrasonogram* OR Ultra-sonogram* OR Echoscop* OR Echo-scop*) | 637,288 |
| #2 | TS=("Roux-en-Y" OR "Cholecystostom*" OR "Choledochostom*" OR "Gastroenterostom*" OR "Jejunoileal Bypass*" OR "Pancreaticojejunostom*" OR "Peritoneovenous Shunt" OR "Portoenterostom*" OR "Gastric Bypass*" OR "Appendectom*" OR "Cholecystectom*" OR "Sphincterotom*" OR "Colectom*" OR "Cecostom*" OR "Colostom*" OR "Duodenostom*" OR "Ileostom*" OR "Jejunostom*" OR "Esophagectom*" OR "Hemorrhoidectom*" OR "Hepatectom*" OR "Liver Transplant*" OR "Pancreas Transplant*" OR "Pancreatectom*" OR "Pancreaticoduodenectom*" OR "Proctectom*" OR “gastrectom*” OR “Gastrostom*” OR “Esophagoplast*” OR “Esophagostom*” OR “Hepatectom*”) | 321,601 |
| #1 | TS=("Machine Learning" OR “machine intelligen*” OR “machine vision*” OR “machine learning” OR “transfer learning” OR “deep learning” OR “neural network*” OR “support vector machine*” OR “automatic segmentation*” OR “Long short term memory” OR “LSTM” OR “supervised learning” OR “unsupervised learning” OR “reinforcement learning*” OR “hierarchical learning*” OR “Image Interpretation*” OR “Prediction model*” OR “image recognition” OR “perceptron”) | 641,632 |

##

## Cochrane (0)

| **Search** | **Query** | **Results** |
| --- | --- | --- |
| #4 | **#1 AND #2 AND #3** | 0 |
| #3 | (ultrasound* OR ultra-sound* OR Echotomograph* OR Echo-tomograph* OR Echotomogram* OR Echo-tomogram* OR Echograph* OR Echo-graph* OR Echogram* OR Echo-gram* OR Sonograph* OR Sono-graph* OR Sonogram* OR Sono-gram* OR Ultrasonograph* OR Ultra-sonograph* OR Ultrasonic* OR Ultra-sonic* OR Ultrasonogram* OR Ultra-sonogram* OR Echoscop* OR Echo-scop*):ti,ab,kw | 51,543 |
| #2 | ("Roux en Y" OR "Cholecystostom*" OR "Choledochostom*" OR "Gastroenterostom*" OR "Jejunoileal Bypass*" OR "Pancreaticojejunostom*" OR "Peritoneovenous Shunt" OR "Portoenterostom*" OR "Gastric Bypass*" OR "Appendectom*" OR "Cholecystectom*" OR "Sphincterotom*" OR "Colectom*" OR "Cecostom*" OR "Colostom*" OR "Duodenostom*" OR "Ileostom*" OR "Jejunostom*" OR "Esophagectom*" OR "Hemorrhoidectom*" OR "Hepatectom*" OR "Liver Transplant*" OR "Pancreas Transplant*" OR "Pancreatectom*" OR "Pancreaticoduodenectom*" OR "Proctectom*" OR “gastrectom*” OR “Gastrostom*” OR “Esophagoplast*” OR “Esophagostom*” OR “Hepatectom*”):ti,ab,kw | 4,302 |
| #1 | ("Machine Learning" OR “machine intelligen*” OR “machine vision*” OR “machine learning” OR “transfer learning” OR “deep learning” OR “neural network*” OR “support vector machine*” OR “automatic segmentation*” OR “Long short term memory” OR “LSTM” OR “supervised learning” OR “unsupervised learning” OR “reinforcement learning*” OR “hierarchical learning*” OR “Image Interpretation*” OR “Prediction model*” OR “image recognition” OR “perceptron”):ti,ab,kw | 5,855 |
